# Relationship Between Obstructive Sleep Apnea and Balance on Computerized Dynamic Posturography

**DOI:** 10.7759/cureus.30973

**Published:** 2022-11-01

**Authors:** Meha G Fox, Helen S Cohen, Haleh Sangi-Haghpeykar, Masayoshi Takashima

**Affiliations:** 1 Otolaryngology - Head and Neck Surgery, Baylor College of Medicine, Houston, USA; 2 Obstetrics and Gynecology, Baylor College of Medicine, Houston, USA; 3 Otolaryngology - Head and Neck Surgery, Houston Methodist Academic Institute, Houston, USA

**Keywords:** sleep disorders, posturography, balance, sleep deprivation, obstructive sleep apnea

## Abstract

Background: Obstructive sleep apnea (OSA) leads to chronic sleep deprivation. The relationship between OSA and balance is poorly understood.

Aim/Objective: This study aimed to determine if OSA adversely affects standing balance.

Material and Methods: Adults with a clinically indicated polysomnogram (PSG) diagnostic of OSA, who were not on therapy, were recruited from an academic tertiary care referral clinic. Subjects completed the Epworth Sleepiness Scale (ESS), the Stanford Sleepiness Scale (SSS), and the STOP-BANG questionnaire (SBQ). Their balance was tested with the Sensory Organization Test (SOT) of computerized dynamic posturography (CDP).

Results: Sixteen subjects participated in the study, including three with mild OSA, six with moderate OSA, and seven with severe OSA. CDP scores were not related to the subjective screening for OSA (ESS, SSS, and SBQ) or to objective measures of OSA (apnea-hypopnea index, respiratory disturbance index, and oxygen saturation nadir).

Conclusion: Subjective and objective measures of sleepiness and sleep disorder are not related to standing balance. The sleep deficit from OSA did not affect standing balance. Therefore, OSA patients are unlikely to be at significant risk for falls due to OSA.

## Introduction

Sleep impairments negatively affect the ability to function well. For example, sleep disturbances can cause cognitive and psychiatric disorders as well as balance deficits, which may cause falls. The most common sleep-related breathing disorder, obstructive sleep apnea (OSA), is characterized by recurrent upper airway resistance and resultant arousal. Chronic, untreated OSA can result in fragmented and poor-quality sleep, which may result in an increased risk of cognitive deficits, hypertension, and cardiopulmonary diseases [1].

An increased prevalence of OSA has been shown to result in higher incidences of occupational accidents [2]. Although excessive somnolence has been suspected, no scientific studies have examined balance deficits related to OSA as being the underlying cause. Some studies have shown impaired balance after acute sleep deprivation. For example, Cuthbertson et al. found significantly decreased computerized dynamic posturography (CDP) scores in otherwise healthy, acutely sleep-deprived post-call resident physicians [3]. The sway-referenced eyes-closed condition, in which the motion of the dynamic force platform is matched to the subject’s own postural sway in order to make kinesthetic input irrelevant while visual input is unavailable, is most consistent with vestibular impairments. Studies of cadet pilots who were sleep deprived for 40 hours [4] and studies of students and other acutely sleep-deprived adults [5,6] have shown similar findings. Morad et al. studied 12 acutely sleep-deprived subjects over a 26-hour period. They showed that a decline in postural testing scores was consistent with increased fatigue and correlated with Stanford Sleepiness Scale (SSS) scores [7]. Avni et al. also demonstrated that the low median sway parameter of CDP was the most affected by fatigue [8]. A technically more sophisticated version of posturography was validated with acutely sleep-deprived young adults [9]. Sleep-deprived farmers have been shown to have increased postural sway in a single-foot stance, with eyes open or closed [10]. None of those studies used subjects with OSA, who have chronic rather than acute sleep deprivation. Therefore, those studies did not indicate the performance of OSA patients on balance tests.

Degache et al. reported that on static posturography, postural sway with eyes open was increased in OSA patients compared to controls [11]. They found an association with two indices on polysomnography (PSG), namely, the apnea-hypopnea index (AHI) and the oxygen desaturation index (ODI) [12]. Kenezaki and Ogawa found that on static postural testing with eyes open, the details of which were not described, OSA patients had increased mediolateral postural sway compared to controls, and this increase was related to an elevated AHI score [13]. The eyes-open condition is not associated with vestibular impairments, so the findings suggest some loss of ability to use visual information to control postural sway.

The goal of the present study was to determine the relationship between OSA and dynamic balance using PSG results and CDP scores as well as to determine if the severity of OSA and the degree of dynamic balance deficits are related.

## Materials and methods

Subjects

Adults with a clinically indicated PSG establishing the diagnosis of OSA (AHI > 5) were recruited from an academic tertiary care referral clinic for OSA, using a convenience sample of patients who were available. Subjects who were non-compliant with continuous positive airway pressure therapy (CPAP) or those who had undergone surgery for OSA but had persistent OSA documented in a postoperative PSG were included. One subject had had a tonsillectomy and uvulopalatopharyngoplasty, seven months prior to the sleep study. Another subject had had an epiglottopexy, two months prior to the sleep study. No subjects took vestibular-suppressant medications.

Patients were excluded for regular use of CPAP; prior oropharyngeal surgical intervention for OSA, including but not limited to tonsillectomy, palatal surgery, and pharyngoplasty with the resolution of OSA; lack of a postoperative PSG; pregnancy; musculoskeletal, vestibular, or neurologic disorders; recent ear infection; a history of neurotologic surgery; and weight over 136 kg. The study was approved by the Institutional Review Board for Human Subjects Research at our institution. Subjects gave written informed consent prior to participation.

Methodology

Office screening tools for OSA include the Epworth Sleepiness Scale (ESS), the SSS, and the STOP-BANG questionnaire (SBQ) [14-18]. A definitive diagnosis of OSA is made by overnight PSG. Measures derived from PSG are the AHI, the respiratory disturbance index (RDI), the oxygen saturation nadir (O_2_ nadir), and the ODI [12]. AHI is defined as the total number of apneic and hypopneic episodes per hour of sleep. AHI of 5-15 is consistent with mild OSA, 15-30 is consistent with moderate OSA, and greater than 30 is consistent with severe OSA. RDI includes AHI and respiratory effort-related arousals (RERAs) that do not meet the criteria for apneas or hypopneas. O_2_ nadir is the lowest level of oxygen saturation during sleep. ODI is the number of times the oxygen level decreases by at least 3% from baseline per hour of sleep.

The subject’s PSG results were recorded, including total sleep time, AHI, RDI, O_2_ nadir, and ODI. The ESS score from the first clinic visit was also recorded. At the time of testing, subjects completed the SSS and SBQ. CDP (Equitest; Natus Medical Inc., Clackamas, Oregon) testing was then completed. CDP is performed on a computerized force platform, which measures the change in postural sway under the subject’s feet. The subject stands in the middle of the force platform, looking toward a screen that surrounds the subject on three sides so the entire visual field is enclosed. The force platform and the surrounding screen can be moved independently. To maintain good hygiene and to standardize testing, subjects wore socks but no shoes. During testing, subjects wore a standard safety harness to prevent falls. Testing in this manner is standard in our laboratory. Subjects were tested using the standard protocol of three trials of each of the six subtests in the Sensory Organization Test (SOT): (1) eyes open, (2) eyes closed, (3) eyes open during sway-referenced motion of the visual surround to render visual input unreliable, (4) eyes open during sway-referenced motion of the force platform plate to render kinesthetic input unreliable, (5) eyes closed during sway-referenced motion of the force platform to render vision unavailable and kinesthetic input unreliable, and (6) eyes open during sway-referenced motion of the visual surround and force platform to render visual and kinesthetic input unreliable. Subjects were allowed to rest in between trials if needed.

We studied independent measures of OSA that are derived from PSG, including AHI, RDI, O_2_ nadir, and ODI. The primary dependent measure was the equilibrium score on the first trial of SOT condition 5 because abnormal scores in this condition are consistent with vestibular impairment. Secondary dependent measures included the score on the first trial of SOT conditions 3, 4, and 6, the composite equilibrium score, and the visual score. The CDP system has a large database of normals. The system compares the test subject to age- and sex-matched data in the database to determine if the score is within the range for normal controls. Therefore, no control group is needed.

Statistical analysis

The relationships between variables of interest were assessed by Pearson correlations or Spearman correlations for non-continuous data. Other associations were assessed by Chi-square or Fisher's exact tests. P-values of less than 0.05 were considered significant. All analyses were performed in Statistical Analysis System (SAS) software version 9.4 (SAS Institute, Cary, North Carolina).

## Results

Study subjects were 16 people, among which eight were females and eight were males; mean age was 52 ± 10 years. The mean body mass index (BMI) was 29.3 ± 6.8 m^2^/kg. Three subjects (18.75%) had mild OSA, six subjects (37.5%) had moderate OSA, and seven subjects (43.75%) had severe OSA based on the American Academy of Sleep Medicine criteria [18]. The mean O_2_ nadir was 83.9 ± 5.3%, and 11 subjects (68.75%) had O_2_ nadir below 88% (Table 1). ODI was only available for three subjects in the study; thus, it was not included in the analysis. CDP scores are listed in Table 2. Conditions 3 and 4 have significantly more normal than abnormal responses, p < 0.05. In conditions 5 and 6, there was no significant difference in the number of normal and abnormal responses.

**Table 1:** Demographics and polysomnography results of cohort BMI: Body mass index; AHI: Apnea-hypopnea index; RDI: Respiratory disturbance index; OSA: Obstructive sleep apnea; F: Female; M: Male; n/a: Not available.

Subject	Age (Years)	Sex	BMI (m^2^/kg)	AHI	RDI	O_2_ Nadir (%)	Severity of OSA
1	42	F	38.4	11.7	22.3	81	Mild
2	60	F	29.1	30.1	42.1	88	Severe
3	54	M	30.1	73.4	n/a	88	Severe
4	50	F	20.5	13.0	23.6	91	Mild
5	38	M	25.7	27.9	n/a	84	Moderate
6	43	M	28.4	27.6	28.8	72	Moderate
7	46	M	26.6	10.8	n/a	91	Mild
8	64	M	27.9	84.2	84.2	85	Severe
9	55	M	30.6	37.5	n/a	91	Severe
10	52	F	29.5	15.7	85.6	83	Moderate
11	50	M	26.2	41.6	53.5	82	Severe
12	57	F	41.6	16.2	18.8	78	Moderate
13	70	F	25.8	21.8	22.8	85	Moderate
14	33	F	44.5	106.6	n/a	78	Severe
15	64	F	21.5	31.4	32.0	84	Severe
16	57	M	23.2	18.3	18.3	82	Moderate

**Table 2:** Computerized dynamic posturography scores SOT: Sensory organization test; C: Condition. Data from trial 1 in conditions 3-6 are shown. * indicates abnormal score.

Subject	SOT C3	SOT C4	SOT C5	SOT C6	SOT Composite Score	Visual Score
1	88	77	70	54	80	84
2	86	72*	47*	0*	64*	83
3	97	44*	84	72	82	74
4	95	71	74	76	82	87
5	84*	71	69	82	84	86
6	86	84	66	81	81	91
7	92	81	71	78	86	94
8	78*	79	0*	29*	68*	86
9	91	79	61	82	76	86
10	95	78	72	51	70	77
11	89	79	63	46*	75	86
12	65*	0*	0*	0*	41*	41*
13	84	81	66	76	83	93
14	84*	78	65	62	79	91
15	93	61*	0*	0*	51*	74*
16	90	66*	49*	20*	61*	67*

The primary analysis showed no correlation between AHI, RDI, O_2_ nadir, and CDP scores on trial 1 of SOT conditions 3,4, 5, or 6, composite equilibrium score, or visual score (Table 3). We then stratified AHI by mild and moderate-severe, O_2_ nadir by ≥88% and <88%, and CDP scores by normal versus abnormal as determined by age-related normative values supplied by the manufacturer. Analyses of these groups revealed no significant differences in CDP scores. There was also no relationship between subjective scores on questionnaires (ESS, SSS, and SBQ) and CDP scores.

**Table 3:** Correlation coefficients of objective sleep measures, sleep questionnaires, and posturography scores (p-value) SOT conditions and data are shown for trial 1. AHI: Apnea-hypopnea index; RDI: Respiratory disturbance index; ESS: Epworth sleepiness scale; SSS: Stanford sleepiness scale; SBQ: STOP-BANG questionnaire; SOT: Sensory organization test; C: Condition.

Measure	AHI	RDI	O_2_ Nadir	ESS	SSS	SBQ	SOT C3	SOT C4	SOT C5	SOT C6	Composite Score	Visual Score
AHI		0.62 (0.11)	-0.11 (0.67)	-0.06 (0.83)	0.32 (0.24)	0.37 (0.18)			-0.10 (0.70)	0.03 (0.91)	0.11 (0.69)	0.19 (0.49)
RDI	0.62 (0.11)		0.16 (0.64)	-0.38 (0.24)	0.56 (0.09)	0.05 (0.89)			-0.10 (0.76)	-0.04 (0.91)	0.06 (0.87)	0.23 (0.50)
O_2_ nadir	-0.11 (0.67)	0.16 (0.64)							0.18 (0.50)	0.16 (0.59)	0.24 (0.37)	0.23 (0.39)
ESS	-0.06 (0.83)	-0.38 (0.24)			0.10 (0.73)	0.08 (0.79)						
SSS	0.32 (0.24)	0.56 (0.09)		0.10 (0.73)		0.04 (0.90)						
SBQ	0.37 (0.18)	0.05 (0.89)		0.08 (0.79)	0.04 (0.90)							
SOT C3	-0.09 (0.73)	0.12 (0.73)	0.46 (0.07)					0.36 (0.17)	0.29 (0.27)	0.56 (0.2)	0.37 (0.16)	0.46 (0.07)
SOT C4	0.74 (0.79)	0.35 (0.3)	0.14 (0.60)				0.36 (0.17)		0.46 (0.07)	0.48 (0.6)	0.67 (0.005)	0.91 (<0.0001)
SOT C5	-0.10 (0.70)	-0.10 (0.76)	0.18 (0.50)				0.29 (0.27)	0.46 (0.07)		0.78 (0.002)	0.84 (<0.0001)	0.54 (0.03)
SOT C6	0.03 (0.91)	-0.04 (0.91)	0.16 (0.59)				0.56 (0.2)	0.48 (0.6)	0.78 (0.002)		0.91 (<0.0001)	0.65 (0.01)
Composite score	0.11 (0.69)	0.06 (0.87)	0.24 (0.37)				0.37 (0.16)	0.67 (0.005)	0.84 (<0.0001)	0.91 (<0.0001)		0.83 (<0.0001)

## Discussion

The CDP scores on subtests that indicate vestibular function and the scores on self-rated measures of OSA were not related. Kayabasi et al. found no differences in static Romberg testing between mild and moderate/severe OSA patients [19]. Similar to our study, static Romberg testing is typically performed with eyes closed. Thus, our finding with dynamic posturography supports that earlier work. These findings suggest that balance without visual input is unimpaired in these OSA patients with chronic sleep disturbance. This finding contrasts with the other studies with OSA patients including the static posturography studies of Degache et al. [11] and Kanezaki and Ogawa [13], but those studies tested subjects with eyes open.

Some reports have suggested relationships with vestibular function. Kayabasi et al. found a significant difference between the subjects with mild and moderate/severe OSA on tests of the vestibulo-ocular reflex (VOR) weakness on bi-thermal caloric testing [19]. Micarelli et al. found significant differences between healthy controls and subjects with OSA on VOR gain on video head impulse testing [20]. Not surprisingly, they found slight correlations between mean oxygen saturation levels on PSG and VOR gain. The present study examined CDP balance results rather than VOR testing, so the VOR test results are not comparable. Similarly, Mutlu et al. showed lower amplitudes on vestibular evoked myogenic potentials (VEMP) in subjects with severe OSA (AHI greater than 70) [21]. No evidence shows a relationship between CDP scores and VEMP scores, so that study is not comparable to the present study either. Micarelli et al. also reported a negative correlation between the power spectra on postural testing, indicating postural sway and oxygen saturation levels. However, the correlation was weak [20]. These studies of vestibular function suggest some effect of chronic sleep deprivation on vestibular function, perhaps at the level of the brainstem, but not on dynamic balance skill. Tests of balance are multifactorial. Although vestibular input plays a role, many other factors influence balance scores. Balance testing does not directly test the vestibular system in the same way that tests the VOR and VEMP. Thus, CDP may not be testing vestibular function in these subjects.

Our study differed from work evaluating balance deficits in acutely sleep-deprived subjects, whose balance deficits are probably due to their decreased ability to integrate sensory and motor information centrally. The present findings suggest that chronically sleep-deprived patients continue to centrally integrate sensory inputs to maintain balance. They may get just enough sleep each night to be able to perform these automatic motor skills, even if cognition is impaired. This idea is supported by the finding that scores on SOT condition 4, in which vision is reliable and not disturbed.

Limitations

The sample size of 16 is relatively small. We were unable to state the statistical power. The low association between the studied measures of balance and OSA suggests that a larger cohort would not have altered the results significantly, although the value of a future study with a larger sample cannot be ruled out. Also, CDP and all other types of balance tests are only indirect measures of vestibular function. We did not perform direct tests of the vestibular system with tests of the VOR. We tested only standing balance but not walking balance. The kinematics of standing and walking balance differ. Subjects might have had subtly impaired walking balance when confronted with environmental challenges that were not evaluated in this study.

## Conclusions

In this study, despite having known chronic sleep deficits, standing balance was not impaired in patients with OSA. Therefore, having OSA is probably not a risk factor for falls in these patients. These findings suggest that subjective and objective tests of sleep disorders are not necessarily related to standing balance. Clinicians who are concerned about falls or imbalance in patients with chronic sleep disorders should probably consider problems other than OSA.
